# Regulation of G2/M Transition by Inhibition of WEE1 and PKMYT1 Kinases

**DOI:** 10.3390/molecules22122045

**Published:** 2017-11-23

**Authors:** Matthias Schmidt, Alexander Rohe, Charlott Platzer, Abdulkarim Najjar, Frank Erdmann, Wolfgang Sippl

**Affiliations:** Institute of Pharmacy, Martin-Luther-University Halle-Wittenberg, Wolfgang-Langenbeck-Str. 4, 06120 Halle (Saale), Germany; alexander.rohe@pharmazie.uni-halle.de (A.R.); charlott.platzer@pharmazie.uni-halle.de (C.P.); a.k.najjar@hotmail.com (A.N.); frank.erdmann@pharmazie.uni-halle.de (F.E.); wolfgang.sippl@pharmazie.uni-halle.de (W.S.)

**Keywords:** G2/M transition, WEE1, PKMYT1

## Abstract

In the cell cycle, there are two checkpoint arrests that allow cells to repair damaged DNA in order to maintain genomic integrity. Many cancer cells have defective G1 checkpoint mechanisms, thus depending on the G2 checkpoint far more than normal cells. G2 checkpoint abrogation is therefore a promising concept to preferably damage cancerous cells over normal cells. The main factor influencing the decision to enter mitosis is a complex composed of Cdk1 and cyclin B. Cdk1/CycB is regulated by various feedback mechanisms, in particular inhibitory phosphorylations at Thr14 and Tyr15 of Cdk1. In fact, Cdk1/CycB activity is restricted by the balance between WEE family kinases and Cdc25 phosphatases. The WEE kinase family consists of three proteins: WEE1, PKMYT1, and the less important WEE1B. WEE1 exclusively mediates phosphorylation at Tyr15, whereas PKMYT1 is dual-specific for Tyr15 as well as Thr14. Inhibition by a small molecule inhibitor is therefore proposed to be a promising option since WEE kinases bind Cdk1, altering equilibria and thus affecting G2/M transition.

## 1. Introduction

Genes encoding for kinases comprise one of the largest families within the human genome and [1], altogether, 539 kinase genes are known so far [2]. Functionally, kinases catalyze the transfer of the γ-phosphate group of ATP to a given acceptor group, which is either serine, threonine, tyrosine, or histidine. Phosphorylation can affect proteins in a number of ways: it acts as a means of activation or inactivation, alters binding to other proteins, or changes subcellular localization. Through the activity of the kinases’ counterparts, the phosphatases, this process is fully reversible, giving this post-translational modification a switch-like character [3]. Therefore, kinases are involved in intertwined networks and feedback loops, most often in a redundant manner, to control cellular functions [4,5].

Besides functional aspects, the molecular structure within the kinase family is highly similar, with the exception of the histidine kinases [6]. The kinase domain of all kinases consists of two lobes: an *N*-terminal lobe, mainly consisting of β-sheets, and a *C*-terminal lobe, dominated by α-helical structural elements. Both parts are linked via a hinge region containing the binding motif for the adenine moiety of ATP. The ribose moiety and the phosphate groups of ATP are coordinatively locked into position by a divalent magnesium ion and a conserved lysine residue [7]. Features differing between kinases, such as the gatekeeper residue and other non-conserved regions, are of major importance for kinase inhibition. Another typical feature of kinases is the activation loop, which contains the conserved DFG motif and is of major importance for the catalytic mechanism.

Generally, there are three ways to inhibit a kinase: substrate-site targeting inhibitors disrupt the protein-protein interaction between the kinase and its direct downstream target. Allosteric inhibitors, sometimes referred to as type III inhibitors, target a site different from the substrate or co-substrate binding site, even though they may bind in spatial proximity to it (reviewed in [8]). ATP-competitive inhibitors displace the co-substrate from its binding site. With respect to the conformation adopted by the conserved DFG motif that controls the kinase activation state [9], ATP-competitive inhibitors can be further divided in two subgroups: type I, type II, and the so-called type I 1/2 inhibitors [10]. Since all kinases utilize ATP as a co-substrate, affinity and selectivity have to be achieved through specific interactions with hydrophobic pockets adjacent to the ATP-binding site [11].

## 2. Physiological Role of WEE Family Kinases

In humans, the WEE kinase family consists of three kinases: PKMYT1 (membrane-associated tyrosine- and threonine-specific cdc2-inhibitory kinase) and two WEE1 kinases (WEE1, WEE1B). Both WEE1 kinases differ in temporal and spatial expression and, in somatic cells, only WEE1 appears to be relevant [12]. Therefore, WEE1B is excluded in the following and only WEE1 and PKMYT1 are included in the term ‘WEE kinases’. The central kinase domain of WEE kinases is atypical; although the tyrosine kinase activity for WEE1 and PKMYT1 is undisputed [13,14], sequence similarity searches do not place them in any of the tyrosine kinase subfamilies, and comparison with the full kinome led to the formation of a separate kinase family consisting of these two kinases [15,16].

WEE1 and PKMYT1 act as cell cycle regulating kinases. The cell cycle is organized into a series of intertwined pathways, whereby the initiation of each event depends upon the successful completion of previous events [16]. Cell division (mitosis) starts the cycle; subsequently, the cells either go into a resting phase (called G0) or a presynthetic (gap) phase (called G1), in which enzyme production occurs in preparation for de novo nucleic acid synthesis. The production of DNA then occurs in an S-phase (synthesis). The S-phase is followed by another gap-phase (G2), in which RNA, critical proteins, and the mitotic spindle apparatus are generated for the next mitotic (M) phase [17]. This ordered progression is guarded by cell cycle checkpoints, i.e., mechanisms by which the cell actively halts progression through the cell cycle until it is ensured that earlier processes, such as DNA replication or mitosis, are completed [18]. In response to endogenous and exogenous sources of DNA damage, these mechanisms are indispensable for maintaining genomic integrity [19]. Activation of DNA damage checkpoints is enabled by the recognition of DNA-damage by sensors, followed by an ordered activation of upstream kinases (ATM (ataxia-telangiectasia mutated)/ATR (ATM- and Rad3-related)) and effector kinases (Chk1 (checkpoint kinase1)/Chk2); the latter can directly target the major cell cycle machinery. A cell cycle arrest or delay upon DNA damage can be induced intra S-phase and at the transitions from G1 to S and from G2 to M-phase [20]. The decision to enter mitosis primarily depends on the activity of cyclin-dependent kinase 1 (Cdk1). Cyclin-dependent kinases are catalytically inactive in their monomeric forms, and their concentrations remain quite constant throughout the cell cycle [21,22]. Association with activators (cyclins) leads to heterodimeric active kinase complexes that can phosphorylate hundreds of downstream targets [23,24]. Cyclins are proteins that oscillate in synchrony with the cell cycle, thereby regulating the activity of the respective Cdk exactly as needed for proper cell cycle progression. In mammalian cells, A- and B-type cyclins are synthesized and degraded around the time of mitosis and are regarded as mitotic cyclins. Importantly, Cyclin B (CycB) accumulation and degradation occurs slightly later than Cyclin A (CycA), regulated at the levels of transcription and proteolysis [25]. After the initiation of the G2/M transition by complexation of CycA and the phosphorylation of various downstream targets [26], CycA is degraded and Cdk1 becomes part of the M-phase promoting factor (MPF), which is composed of Cdk1 and CycB [27,28,29]. Cdk1/CycB is, in turn, regulated by complex mechanisms. In the inner feedback loops, Cdk1/CycB activity is controlled by the balance between WEE kinases and Cdc25 phosphatases, which are responsible for the status of inhibitory phosphorylation at Thr14 and Tyr15 of Cdk1 kinase [30]. These kinases and phosphatases are in turn regulated by Cdk1 activity. Once activated, Cdk1/CycB can phosphorylate WEE1 and PKMYT1 to promote their inactivation via different cascades [31,32,33]. Additionally, Cdk1/CycB can activate Cdc25 phosphatase, which shifts the equilibrium even more towards active Cdk1/CycB (autoamplification). In other words, through the inner feedback loops, Cdk1/CycB can stimulate its further activation by directly activating its activators and deactivating its inhibitors. The regulating network becomes even more complex if outer feedback loops, i.e., indirect regulation mechanisms besides Thr14/Tyr15 phosphorylation, are taken into account. These feedback loops are superimposed on the inner feedback loops but act via other mediating enzymes such as Plk1 (polo-like kinase 1). Plk1, a direct and indirect target of Cdk1/CycB, can mediate indirect inhibition through the phosphorylation of WEE kinases and, at the same time, activate the Cdc25 phosphatases [34]. These feedback loops not only promote an efficient activation of Cdk1/CycB, but also ensure that other regulatory factors needed for successful cell division are activated in a coordinated manner. Supporting this notion, short-circuiting the inner feedback loop by the expression of a WEE1/PKMYT1-insenstitive Cdk1 mutant (T14A, Y15F) leads to abnormal cell division [35]. Owing to the numerous feedback loops, Cdk1/CycB activation is considered a bistable process, meaning the majority of the complexes are inactive, active, or approaching one of these states [36,37,38]. Therefore, a cell will or will not enter mitosis, but cannot rest in an intermediate state. Since the inactivation threshold requires lower Cdk1/CycB concentrations than the activation threshold (hysteresis), successful mitotic entry is ensured and, at the same time, the cell is given an opportunity to block mitotic entry in case of premature activity fluctuations [39]. Another important matter in Cdk1/CycB control and mitotic entry is nucleocytoplasmic shuttling. With respect to various posttranslational modifications, subcellular trafficking of CycB and Cdk1/CycB is altered because modifications affect the affinity towards transport proteins that mediate traffic between the nucleus and cytoplasm. Therefore, spatial sequestration can also prevent protein interactions if the requirements for cell cycle progression have not yet been met [40,41]. Altogether, entry into mitosis is controlled not only by the regulation of CycB accumulation, but also by inner and outer feedback loops as well as spatial and time-dependent sequestration of the respective proteins.

**Figure 1:**
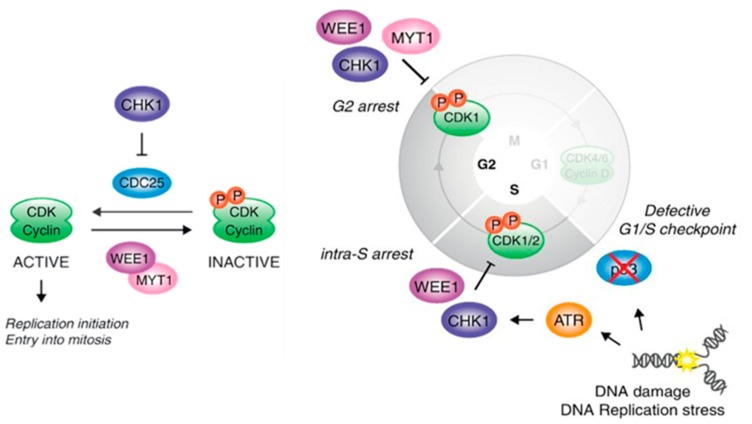
Cell cycle control: DNA damage checkpoint (modified from Aarts et al. [55]). Reproduced with permission from Nick Turner, Current Opinion in Pharmacology; published by Elsevier, 2013.

WEE1 and PKMYT1 are involved in some of these mechanisms showing in Figure 1. WEE1 (1987) and PKMYT1 (1995) were discovered as kinases that are responsible for inhibitory Cdk1 phosphorylation [42]. The kinase activity towards Thr14 and Tyr15 of the Cdk1 protein is high in interphase and decreases due to hyperphosphorylation in M-phase. In contrast to WEE1, PKMYT1 exhibits more restricted substrate specificity, in that it phosphorylates Cdk1 but not Cdk2 complexes. The direct Cdk1/CycB regulation consists of two independent mechanisms. First, inhibitory phosphorylation occurs at Thr14 and Tyr15 [43,44]. Importantly, the phosphorylation of Cdk1/CycB at Thr161 of the Cdk subunit by Cdk activating kinase (CAK) is a prerequisite for the activation of the Cdk/Cyc complex [45,46]. The phosphorylation of Thr161 is, in turn, tightly coupled to Thr14 phosphorylation [47]. Cdk1/CycB is continuously shuttling but mostly cytoplasmic, due to its more active nuclear export [26,48]. Upon binding to CycB, unphosphorylated Cdk1 can be immediately phosphorylated at Thr14 and/or Tyr15 by WEE1 (mostly located to the nucleus, but also present in the cytoplasm to a lesser extent [49]) and/or PKMYT1, but not nuclear CAK. In the absence of Thr161 phosphorylation, these Cdk1/CycB complexes are unstable and therefore release monomeric Cdk1 with its various possible phosphorylation patterns [50]. Nuclear trafficking pathways addressing pThr14-Cdk1/CycB are probably responsible for the observation that Thr161 modification is strictly associated with Thr14 modification. The actual activation is then mediated by nuclear Cdc25 phosphatases that hydrolyze the inhibitory phosphorylation while maintaining the required Thr161 modification. The tight coupling of Thr161 and Thr14 phosphorylation protects Cdk1/CycB from premature activation and ensures that it is only activated by dephosphorylation. As a second mechanism to regulate Cdk1/CycB, there is a *C*-terminal domain within the PKMYT1 protein that interacts with Cdk1 complexes [51]. In contrast to the mostly nuclear WEE1, PKMYT1 is localized to the endoplasmic reticulum and Golgi complex by a membrane-tether. The binding of Cdk1 complexes by PKMYT1 sequesters them in the cytoplasm, thereby precluding entry into the nucleus and preventing cell cycle progression. Overexpression of PKMYT1 prevented entry into mitosis, but the catalytic kinase activity was not essential for a cell cycle delay observed in human cells. Although the accumulation within the G2/M population was less efficient for catalytically inactive PKMYT1 than wild-type PKMYT1, the importance of the direct protein-protein interaction should not be underestimated. These findings may partly explain the limited effects on mitotic entry of PKMYT1 knockdown by RNA interference and highlight the need for small molecule inhibitors [52,53,54].

When DNA damage occurs to a cell in G2-phase, the subsequent cell cycle arrest is achieved through posttranslational modifications. Upon repair of the damaged DNA, the cell can resume the cell cycle, a process that is referred to as checkpoint recovery [56]. Most recently, it was suggested that PKMYT1 plays a relatively minor role in unperturbed cell cycle and rather bears an essential function in G2 checkpoint recovery [57]. This PKMYT1 function in checkpoint recovery is completely independent of WEE1. It is hypothesized that the G2 DNA damage checkpoint maintains Cdk1 in a Thr14- and Tyr15-phosphorylated, inactive state, which is controlled by enhanced WEE kinase activity and reduced Cdc25 phosphatase activity. As Plk1 is an important regulator of G2 checkpoint recovery, it mediates the activation of Cdc25 and the inactivation of WEE1. Because PKMYT1 is known to be negatively regulated by Plk1 through direct phosphorylation, as well as found to be inactivated during checkpoint recovery, there might be a connection [31]. The downregulation of WEE1 and PKMYT1 was found to accelerate checkpoint recovery and mitotic entry.

## 3. Structural Analysis of WEE Family Kinases

**Figure 2:**
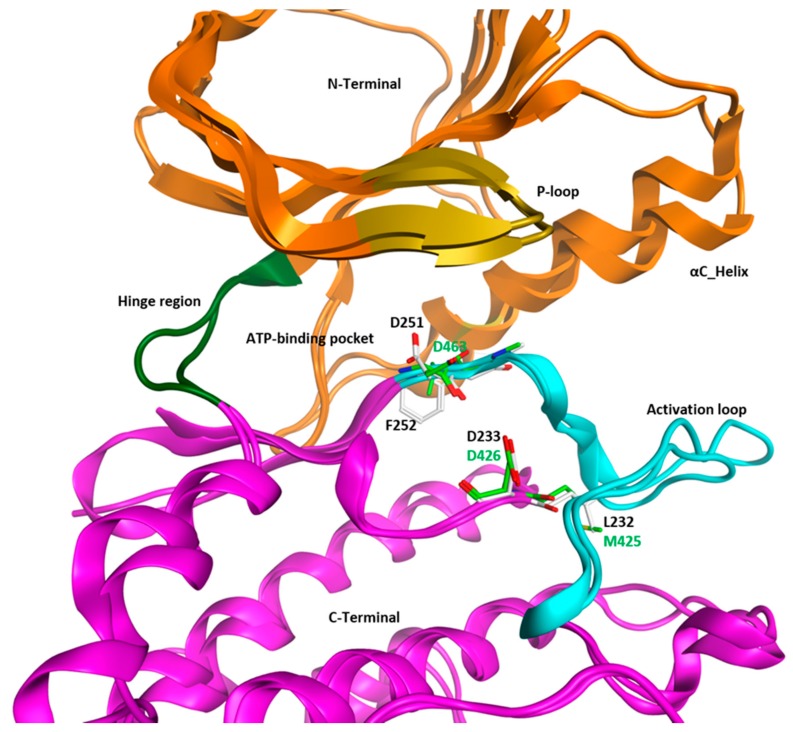
Superposition of crystal structures from the Protein Data Bank (PDB) of WEE1 (PDB ID: 1X8B) and PKMYT1 (PDB ID: 3P1A). The protein ribbon is colored as following: *N*-Terminus: gold; *C*-Terminus: magenta; hinge region: dark green; activation loop: cyan; P-loop: yellow; PKMYT1 residues: white; WEE1 residues: green.

The kinase domain of WEE1 has been co-crystallized with several pyrrolocarbazoles and pyrroloindoles, which are available in the Protein Data Bank (PDB IDs: 3CQE, 3CR0, 2Z2W, 3BIZ, 3BI6, 2IN6, 2IO6, and 1X8B). Recently, Zhu et al. reported six crystal structures of the WEE1 kinase domain co-crystallized with several kinase inhibitors: MK1775, Bosutinib, a Bosutinib-isomer, PD-166285, PHA-848125, and PF-03814735 (PDB IDs: 5V5Y, 5VC3, 5VC4, 5VC5, 5VC6, and 5VD2, respectively) [58]. Additionally, the X-ray structure of WEE1B in complex with the inhibitor MK1775 is available (PDB ID: 5VDK). Until recently, there was only one crystal structure of PKMYT1 in apo-form (PDB ID: 3P1A) available in the Protein Data Bank. In the work of Zhu et al., eight crystal structures of PKMYT1 kinase domain (PDB IDs: 5VCV, 5VCW, 5VCX, 5VCY, 5VCZ, 5VD0, 5VD1, and 5VD3) in complex with known inhibitors (Dasatinib, Pelitinib, Saracatinib, Bosutinib, Bosutinib-isomer, MK1775, PHA-848125) were published [58]. The overall structure of the WEE family kinases is similar to other known kinase structures, which consists of two lobes connected by a hinge region, as seen in Figure 2. The catalytic domain is located between both terminal lobes and shows high conservation. The *N*-terminal lobe is formed by five standard β-sheets and one α-helix, called αC-helix, surrounding the ATP-binding cleft (Figure 2). The *N*-terminal domain contains a flexible Glycine-rich loop, called P-loop or G-loop, which adopts different conformations depending on the catalytic state and the bound ligand. The P-loop forms the top-surface of the ATP-binding pocket. The *C*-terminal domain is composed mainly of α-helices and contains the activation loop (Figure 2). Within the *C*-terminal domain, the catalytic cleft is divided into a front cleft, which involves the ATP-binding pocket, and a back cleft, including the important residues for kinase regulation. The back cleft contains a catalytic segment that includes the essential catalytic aspartate (WEE1: Asp426, PKMYT1: Asp233) (Figure 2) and the activation loop which can undergo conformational changes. Since the residue preceding the catalytic aspartate is not an arginine but rather nonpolar (WEE1: Met425, PKMYT1: Leu232) (Figure 2), it has been suggested that WEE kinases do not need to be activated by the phosphorylation of the activation loop [59]. The conserved Asp-Phe-Gly (DFG motif; WEE1: Asp463, Leu464, Gly465; PKMYT1: Asp251, Phe252, Gly253), which plays a role in the regulation of the activity state of kinases, is located in the catalytic segment, specifically at the beginning of the activation loop. The reported crystal structures of WEE1 and PKMYT1 are found in the active state with a DFG in conformation. In this conformation, the DFG aspartate is moving towards the ATP-binding pocket. It is noteworthy that WEE1 possesses a leucine residue instead of a phenylalanine in the DFG motif in comparison to PKMYT1 (Figure 2).

**Figure 3:**
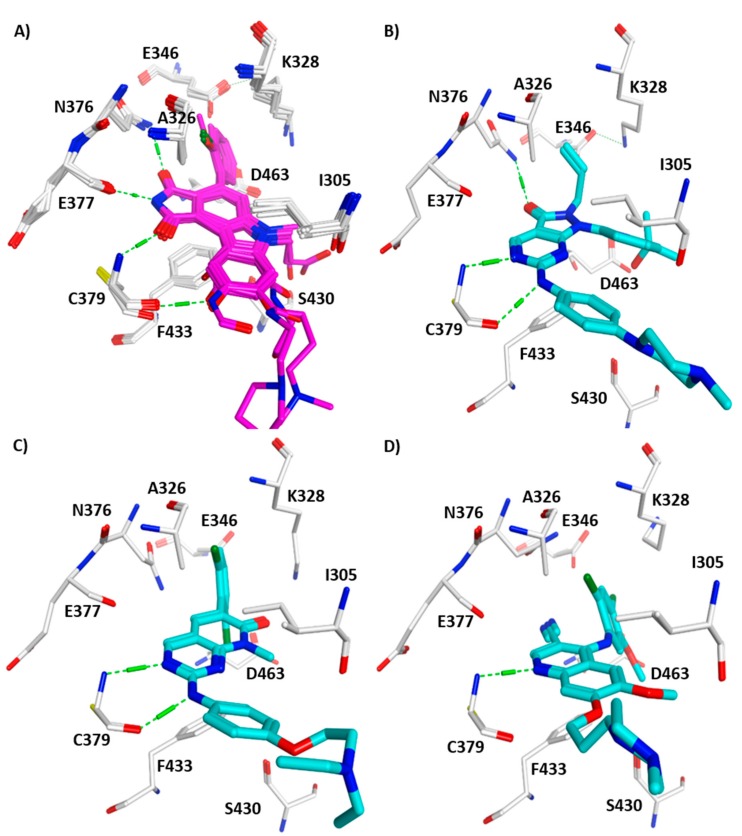

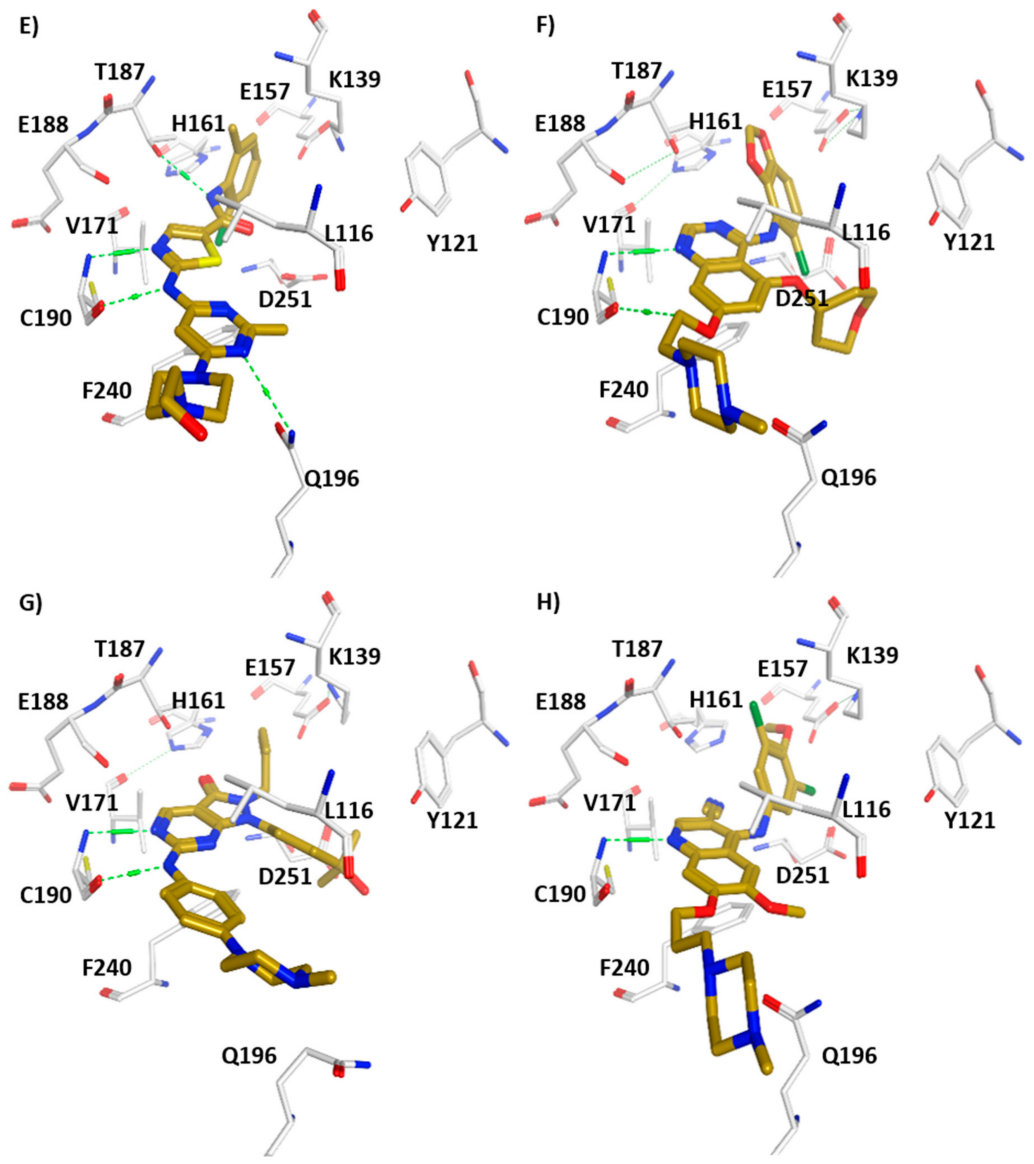
Interaction of WEE1 and PKMYT1 inhibitors observed in reported crystal structures. Hydrogen bonds with hinge region residues are displayed as dashed green lines. (**A**) Superposition of the X-ray structures of WEE1 in complex with pyrrolocarbazoles and pyrroloindoles (colored magenta); (**B**) WEE1 in complex with the diaminopyrimidine derivative MK1775 (colored cyan, PDB ID: 5V5Y); (**C**) WEE1 in complex with the pyridopyrimidine derivative PD-0166285 (colored cyan, PDB ID: 5VC5); (**D**) WEE1 in complex with Bosutinib (colored cyan, PDB ID: 5VC3); (**E**) PKMYT1 in complex with Dasatinib (colored yellow, PDB ID: 5VCV); (**F**) PKMYT1 in complex with Saracatinib (colored yellow, PDB ID: 5VCX); (**G**) PKMYT1 in complex with the diaminopyrimidine derivative MK1775 (colored yellow, PDB ID: 5VD0); (**H**) PKYMT1 in complex with the 4-aminquinoline derivative Bosutinib Isomer (colored yellow, PDB ID: 5VCZ).

The structural superposition of the WEE1 kinases co-crystallized with pyrrolocarbazoles and pyrroloindoles shows an almost identical conformation of the kinase domain and a similar binding mode of the bound inhibitors (RMSD; root-mean-square deviation of the backbone atoms is 0.23 Å). Also, the crystal structures of WEE1 in complex with other inhibitors recently published [57] show a high structural similarity and the typical interaction of the inhibitors with the hinge region (Figure 3A–D). Superimposing the crystal structures of apo and complexed PKMYT1 also shows a high structural similarity with only slightly changes of the ATP-binding pocket (Figure 3E–H).

**Figure 4:**
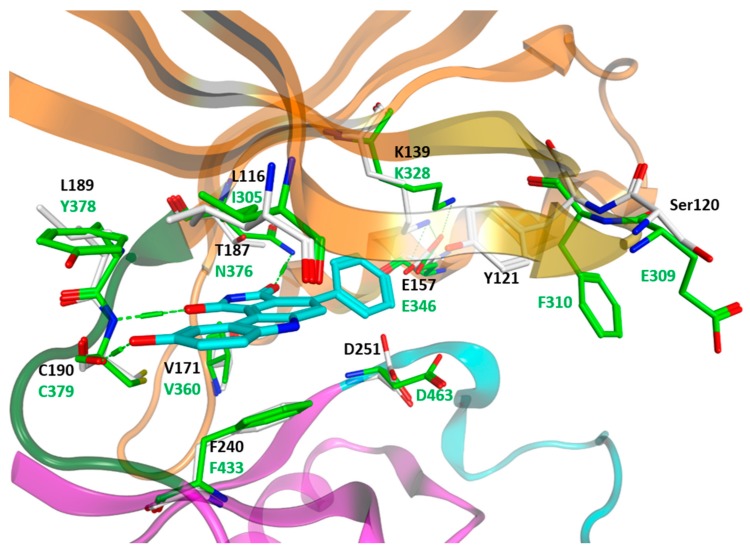
Superposition of the ATP-bindings pocket of WEE1 (PDB ID: 1X8B, green) and PKMYT1 (PDB ID: 3P1A, white residues). Ribbons have been colored as mentioned in Figure 2. The co-crystallized inhibitor PD0407824 of WEE1 is shown in cyan. Hydrogen bonds are displayed as dashed lines.

Superimposing the crystal structures of WEE1 (1X8B) and PKMYT1 (3P1A) results in an RMSD deviation of the backbone atoms of 1.87 Å. The major deviation can be found in the flexible αC-helix and in the activation loop residues (Figure 2). The kinase domain of both kinases shows a sequence identity of 36.5% and a sequence similarity of 53.6% underlying the similarity of both kinases. As previously mentioned, the ATP-binding pocket is located in the front cleft of the kinase domain. Both kinases show only a few mutations in the ATP-binding pocket (Figure 4). The key residues, which form the ATP-binding pocket, are displayed in Figure 2. The overall comparison covering 20 residues within the ATP-binding pocket of WEE family kinases reveals high similarity (similarity: 76.2%, identity: 61.9%, RMSD deviation of the backbone: 0.88 Å). Notably, the bulky gatekeeper residue in WEE1 (Asn376) results in a more restricted back pocket and prevents the interaction of the inhibitors within this pocket. In contrast, PKMYT1 has a small gatekeeper (Thr178), allowing access to the back hydrophobic pocket (Figure 4). Flexible aromatic amino acids are located at the top of the P-loop (WEE1: Phe310, PKMYT1: Tyr121), which adopt different conformations based on the binding state. In the apo-form of PKMYT1, Tyr121 is flipped into the ATP-binding pocket; meanwhile, binding of an inhibitor in WEE1 results in a flip-out conformation of Phe310 (Figure 3). The conserved cysteine residue of the WEE family hinge region (WEE1: Cys379, PKMYT1: Cys190) forms hydrogen bonds with ATP or the bound inhibitors (Figure 4).

Sequence alignment of WEE family members of various species suggests several conserved features across the whole kinase domain [60]. In addition, superimposition studies for WEE1 revealed the closest structural matches to be Ser/Thr kinases, including the active forms of Cdk2 and Chk1. Notably, a tyrosine kinase is not encountered until the 14th top-ranked hit [59]. Besides the structural similarities, the sequence of WEE kinases is more closely related to Ser/Thr kinases than to Tyr kinases, as illustrated by a simple Basic Local Alignment Search Tool (BLAST) comparison of the kinase domains [61]. Taken together, WEE kinases may have evolved from Ser/Thr kinases, and a few key mutations may have converted them to functional Tyr kinases. This hypothesis is supported by the fact that PKMYT1, as a dual-specific kinase, has been observed to phosphorylate Tyr and Thr residues [44]. Both WEE kinases target the same site—the glycine rich loop of Cyclin-dependent kinase 1 (Cdk1). WEE1 acts specifically to phosphorylate Tyr15, while PKMYT1 is dual-specific for Tyr15 as well as Thr14 [50]. Considering their close relationship, where does the difference in substrate specificity come from? For Tyr15 phosphorylation, the features that allow the correct orientation of the substrate are given in both WEE kinases. Meanwhile, a single differing residue in their P-loop may account for the Thr kinase activity of PKMYT1; Thr14 modification requires the substrate to approach the P-loop of the phosphorylating kinase more closely. In WEE1, Glu309 at the top of this loop may hinder a closer approach in the same way that the phosphorylation of Cdk1 is believed to interfere with substrate binding through steric hindrance (Figure 4) [62]. Glu309 in WEE1 is replaced by a serine residue in PKMYT1 (Ser120), which is less bulky and does not cause electrostatic repulsion as does the negative charge of the glutamate side chain (Figure 4). Therefore, in contrast to WEE1, a closer substrate approach is realized that may enable an effective threonine phosphorylation.

## 4. WEE1 and PKMYT1 as Potential Drug Targets in Cancer Therapy

Mutations to p53, a protein of major importance to the G1 checkpoint, have been implicated in more than half of all human oncogenesis [63]. Due to mutations in the p53 network, many cancer cells have defective G1 checkpoint mechanisms [64], which can result in increased DNA damage at the G2 checkpoint compared to normal cells [65]. Selective G2 checkpoint abrogation, disrupting a signal pathway not involved with p53, should not harm normal cells because they have another, p53-dependent pathway to halt the cell cycle at this point [66]. Therefore, a novel strategy of selective sensitization evolved, combining checkpoint abrogation with DNA damaging agents or radiation [67,68]. The first (unintentionally) used G2 checkpoint abrogator was caffeine; the exact mechanism, however, still remains unclear [69,70]. Abrogation of the G2 checkpoint forces cells with unrepaired DNA damage into premature mitosis. This checkpoint abrogation can be induced by pharmacological manipulation, resulting in mitotic catastrophe and apoptosis when the extent of unrepaired DNA damage exceeds a varying threshold [71,72]. Checkpoint abrogation is a prerequisite for mitotic catastrophe [73], which results in apoptotic and non-apoptotic cell death [74]. Yet, apoptosis is not required for the lethal effect of mitotic catastrophe [75]. Cells with intact G1 checkpoint arrest, such as normal cells or cancer cells with intact p53 signaling, are less dependent on the G2 checkpoint arrest and are, therefore, not as sensitive towards G2 checkpoint abrogation [76]. Inhibitory Cdk1 phosphorylation is responsible for radiation-induced G2 arrest, and this checkpoint can be abrogated by expressing a non-phosphorylatable Cdk1 mutant [77]. Confirmed or suggested targets for G2 checkpoint abrogation and mitotic catastrophe are WEE1, PKMYT1, Chk1, and Hsp90 (heat shock protein 90) [78,79]. Indeed, selective WEE1 inhibition by e.g., MK1775 showed promising effects, just as predicted [80].

**Figure 5:**
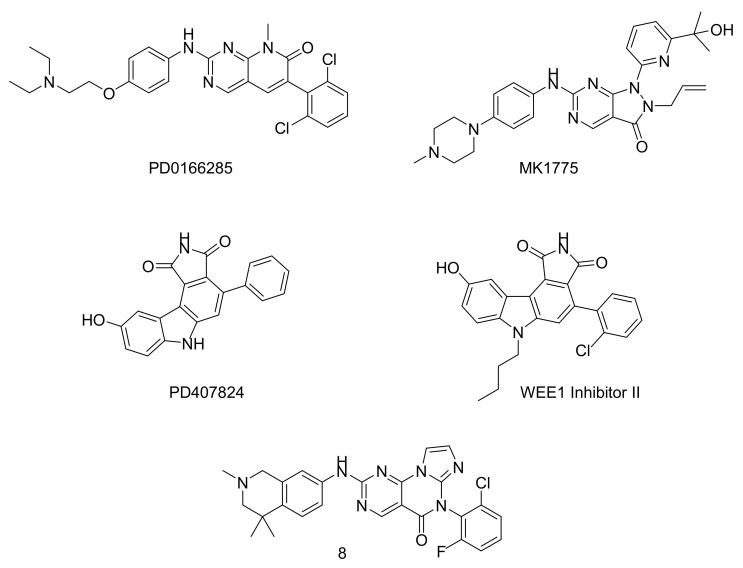
Chemical structure of WEE1 inhibitors.

For WEE1, five compounds reviewed as inhibitors are represented in Figure 5 [81]. PD0166285 is the first compound that has been reported with an inhibitory activity against WEE1. It is a non-selective 6-aryl-pyrido[2,3-*d*]pyrimidine derivative active in a nanomolar range (IC_50_ = 24 nM) on WEE1, but is also active on PKMYT1 and a range of other kinases including c-Src (*C*-terminal Src kinase), EGFR (epidermal growth factor receptor), FGFR1 (fibroblast growth factor receptor 1), CHK1, and PDGFRb (Beta-type platelet-derived growth factor receptor) [82]. PD0407824, a staurosporine analogue, with an IC_50_ of 97 nM on WEE1 was more active on CHK1. Optimization of PD0407824 resulted in the discovery of WEE1 inhibitor II, which bears two additional substituents (Figure 4). This compound is more selective against CHK1; however, it showed no activity in cells due to its low solubility and cell permeability. Optimization of WEE1 inhibitor II was attempted, but led to no significant improvement. MK1775 is the first highly potent and selective WEE1 inhibitor (IC_50_ = 5 nM). It was discovered by Merck laboratories through the optimization of an high troughput screening (HTS) hit. MK1775 activity is selective towards p53 defective cells and has no effect on p53^+^ cells. MK-1775 is the most potent and highly selective inhibitor of WEE1 and has recently reached phase I clinical trials. Lastly, a pyrimidine-based tricyclic molecule was proposed in 2015 by AbbVie for the inhibition of WEE1. This molecule (**8**) was designed by a rational hybridization between MK1775 and other WEE1 inhibitors as a most promising molecule, rendering this compound promising for further preclinical evaluation.

**Figure 6:**
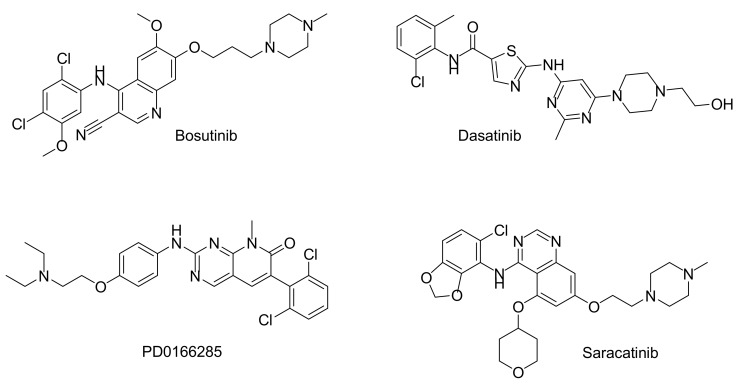
Chemical structures of potent PKMYT1 inhibitors.

For PKMYT1, only nine inhibitors were published as active, and included among them were drugs like the tyrosine kinase inhibitors Dasatinib (IC_50_ = 130 nM) and Bosutinib (IC_50_ = 350 nM), as well as the pyridopyrimidine derivatives PD0166285 (IC_50_ = 7.2 nM), PD1739525, PD1739552, and PD1809705 [83]. Furthermore Pelitinib, Saracatinib, and Tyrphostin AG14784 seem to be weak inhibitors (Figure 6). When comparing the number and strength of PKMYT1 inhibitors with other kinases, PKMYT1 appears to be restricted and difficult to inhibit. This is particularly evident by the selectivity score for PKMYT1 given by Davis et al., with 0.0417 at 3 μM inhibitor concentration [84]. Thus, only 4.17% of the tested compounds inhibit PKMYT1—a fact that is true for only 2% of all tested kinases generally. None of the potential PKMYT1 inhibitors reached clinical trials until now.

### 4.1. Assays

One of the essential tools used to develop new kinase inhibitors consists of detecting the interaction of a test compound with the kinase. There are two possibilities to investigate: first, the determination of the binding affinity of the compound; and second, the detection of kinase inhibition. Usually a high binding affinity correlates with a strong inhibition of kinase function, but this is not always the case.

To detect the binding affinity of a PKMYT1-inhibitor, different binding assays are available, which either use the PKMYT1-full length or PKMYT1-domain. The time-resolved fluorescence resonance energy transfer (TR-FRET)-based binding assay uses the PKMYT1-full length and measures the FRET to a fluorescently labeled ATP-competitive compound that binds to PKMYT1. The FRET is possible upon excitation of a europium labeled antibody, which binds to the kinase. The TR-FRET assay allowed the first inhibitor binding studies of PKMYT1 kinase [83]. To extend the assay range, a fluorescence anisotropy-based binding assay was established in 2014, using the PKMYT1 domain and a fluorescently labeled ATP-competitive compound [85]. Using the two assays led to the identification of nine PKMYT1 inhibitors. In accordance with PKMYT1, a TR-FRET assay for WEE1 is commercially available (LanthaScreen^®^ Eu Kinase Binding Assay, Invitrogen, Carlsbad, CA, USA). The establishment of binding assays for PKMYT1 and WEE1 were part of an analysis of kinase inhibitor selectivity reported by Davis M. et al. [84]. Besides the binding affinity of a compound, the influence on enzyme reaction is more important. In kinase assays, one generally measures the phosphorylation of a substrate with different methods; e.g., scintillation proximity assays (SPAs), homogeneous time-resolved fluorescence (HTRF) assays, fluorescence polarization (FP) assays, Alphascreen assays, radioactive filter assays, or ELISAs [86]. The identification of peptidic substrates for the PKMYT1 kinase in 2015 was the beginning of the development of an FP-based PKMYT1 activity assay, which would allow the first medium throughput screening of PKMYT1 inhibitors [87]. The alternative test systems, e.g., ADP-Glo^TM^ kinase-assay from Promega, determine the unspecific decrease of ATP during the phosphorylation reaction. The commercially available PathScan^®^ Phospho-CDK1 (Tyr15) Sandwich ELISA Kit (Cell Signalling Technology, Danvers, MA, USA) and Phospho-CDK1 (Thr14) Colorimetric Cell-Based ELISA Kit (Aviva Systems Biology, San Diego, CA, USA) are colorimetric detection systems.

In the field of WEE1, research is clearly more progressed. Hirai et al. used a radioactive assay to detect the IC_50_ value of the first selective WEE1 inhibitor MK1775 [79]. The important discovery of MK1775 was a significant step, which boosted the WEE1 kinase investigations. Different cell-based assays were implemented during the development of new WEE1 inhibitors. For example, a cellular ELISA assay was used to determine pyrimidine-based tricyclic compounds as WEE1 inhibitors [88]. Furthermore, cell viability assays [89,90], cytotoxicity assays [91], flow cytometry [81], and p-histone H3 assays were used to investigate the different effects of WEE1 inhibitors in cell lines. The extensive investigation of MK1775 with cell assays was the basis of the following clinical studies.

Generally, the adaption of the test results on isolated enzymes to the effects on cell lines is important to allow an indication of in vivo characteristics of the kinase inhibitor. For WEE1 kinase, this is possible with a lot of different assays, but for PKMYT1 it is not the case. The development of cell-based PKMYT1 assays is still pending and will extend the assay range, which will open up further opportunities to investigate new PKMYT1 inhibitors.

### 4.2. Cell Experiments

MK-1775-treated cells expressing short hairpin RNA against p53 were much more sensitive towards gemcitabine, carboplatin, and cisplatin. In accordance with these findings, the effects of MK1775 monotherapy were only moderate. However, MK1775 enhanced the cytotoxic effects of 5-fluorouracil (5-FU) in p53-deficient colon cancer cells and pancreatic cancer cells, but not in wild-type p53 colon cancer cells [92]. Similar results were obtained in combination with gemcitabine [93], doxorubicin, carboplatin, and cisplatin. Similar to WEE1 knockdown, PKMYT1 knockdown increased the kinetics of G2/M transition, promoted early entry into mitosis, or led to total checkpoint abrogation [53,54]. Doxorubicin-induced G2 arrest in HeLa cells was abrogated when PKMYT1 was knocked down. Downregulation of PKMYT1 shortened the time between checkpoint abrogation and mitotic entry, which also increased the level of subsequent cell death. Therefore, both WEE1 and PKMYT1 may be useful targets for anti-cancer therapy. In xenograft models, PKMYT1 was revealed to be a particularly attractive target because it is of relatively minor importance for normal cell cycle progression. Inhibitors of cell cycle components which are essential for normal cell cycle progression may be too toxic for normal cells and unsuitable for use in therapies. So far, some other findings stand on their own, but highlight the potential for PKMYT1 inhibition in cancer therapy: PKMYT1 was upregulated by more than 10-fold in seven tested ovarian cancer cell lines [94]; and gastric cancer cells were found to overexpress PKMYT1 in response to the α-emitter Bi213 prior to cell death [95]. In an RNAi screening across the entire kinome in combination with cytarabine in leukemias, Chk1 and PKMYT1 were the strongest sensitizers [89]. However, since a selective PKMYT1 inhibitor was not available, MK1775 was used instead to inhibit WEE1 as the most closely related kinase. Leukemia cells were 97-fold more sensitized compared to cytarabine control, raising expectations with regard to PKMYT1 inhibition in future cancer therapy.

### 4.3. Clinical Trials with PKMYT1 and WEE1 Inhibitors

Unfortunately, no specific PKMYT1 kinase inhibitors are known and therefore no clinical studies could be started yet. However, there are 29 clinical trials with the WEE1 inhibitor MK1775 (also known as AZD1775), which test its ability to impair alone or in combination with other cytostatic drugs (e.g., cisplatin, paclitaxel, 5-fluorouracil, and topotecan) the growth of different kinds of cancer (Table S1). At the moment, two studies are still ongoing and five are terminated. To date, only the preliminary results of three terminated projects are published online. Study NCT02196168 was terminated due to inadequate accrual rate. The number of patients was too small and no subject could be recruited for the arm II of the study (placebo + cisplatin). Therefore, no valid data could be obtained. AstraZeneca-sponsored clinical trial NCT02087241 was also stopped earlier. A total of 14 subjects were enrolled in four cohorts with only one to seven patients. During the 13-month observation period, five subjects died and one cohort was extinguished completely. However, the particular reasons which led to the termination decision are unknown. NCT02087176, the third study with 32 participants that was also supported by AstraZeneca, was terminated before data collection and analysis were finished. Probably the weak response rate of 9.4% in the MK1775 + docetaxel group resulted in the decision to abort further investigations. Still, the results of other current studies are awaited by the community, held in suspense and in the hope of obtaining a new drug that makes the cancer therapy more effective.

Table S1 (Supplementary Materials) provides an overview of current and already completed clinical trials with the WEE1 inhibitor MK1775 (AZD1775) (last update 22 June 2017).

## 5. Summary and Perspectives

WEE1 and PKMYT1 are kinases that play pivotal roles in DNA-damage recovery. They are members of the WEE family of kinases and negatively regulate the cell cycle via the phosphorylation of CDK1. Both kinases are considered as main gatekeepers of the G2 cell-cycle checkpoint; their inhibition is particularly effective in cells with deficient p53 signaling. WEE1 and PKMYT1 inhibition, which targets G2/M DNA-damage recovery, acts as combination therapy and represents a target for the radiosensitization of various types of cancers. In this context, both kinases introduced above appear as particularly relevant targets to develop therapeutic agents able to modulate the DNA checkpoint response in cancer cells.

Indeed, binding, activity, and functional studies demonstrate that WEE1 and PKMYT1 are worthwhile targets for further clinical investigations. With MK1775, a potent inhibitor was validated for first clinical trials. Purified recombinant full-length proteins and kinase domain constructs differ substantially in phosphorylation states and catalytic competency, suggesting complex mechanisms of activation. A series of crystal structures reveal unique features that distinguish WEE1 and WEE1B from PKMYT1, and establish the structural basis of differential inhibition by the widely used WEE1 inhibitor MK1775. Several previously unrecognized inhibitors of WEE kinases were discovered and characterized.

## Supplementary Materials

Supplementary materials are available online. Table S1: Clinical trials summary.
