# Supplementary material for: Regulation of G2/M Transition by Inhibition of WEE1 and PKMYT1 Kinases
Source: Molecules. 2017 Nov 23;22(12):2045. doi: 10.3390/molecules22122045 (PMC6149964; doi:10.3390/molecules22122045)
Supplement: Supplementary file 1 [file molecules-22-02045-s001.pdf]

Clinical Trial Identifier	Title	Cancer	Drug	Phase	Status
NCT01164995	**Study With Wee-1 Inhibitor MK-1775 and Carboplatin to Treat p53 Mutated Refractory and Resistant Ovarian Cancer**	Epithelial Ovarian Cancer	MK1775 + Carboplatin	2	unknown
NCT01047007	**A Dose Escalation Study of MK1775 in Combination With 5-FU or 5-FU/CDDP in Patients With Advanced Solid Tumor**	Solid Tumors	MK1775 + 5-FU vs.<br>MK1775 + 5-FU/Cisplatin	1	terminated
NCT02095132	**WEE1 Inhibitor MK-1775 and Irinotecan Hydrochloride in Treating Younger Patients With Relapsed or Refractory Solid Tumors**	Childhood Central Nervous System Neoplasm; Recurrent Malignant Solid Neoplasm	MK1775<br>MK1775 + Irinotecan	1<br>2	recruiting
NCT01076400	**A Study of MK1775 in Combination With Topotecan/Cisplatin in Patients With Cervical Cancer**	Cervical Cancer	MK1775 + Topotecan/Cisplatin<br>Topotecan/Cisplatin ± MK1775	1<br>2	terminated
NCT00648648	**A Dose Escalation Study of MK1775 in Combination With Either Gemcitabine, Cisplatin, or Carboplatin in Adults With Advanced Solid Tumors**	Solid Tumors	MK1775 vs.<br>MK1775 + Gemcitabine vs.<br>MK1775 + Cisplatin vs.<br>MK1775 +Carboplatin	1	completed

NCT01357161	**A Study of MK-1775 in Combination With Paclitaxel and Carboplatin Versus Paclitaxel and Carboplatin Alone for Participants With Platinum-Sensitive Ovarian Tumors With the P53 Gene Mutation**	Ovarian Cancer	Pacitaxel/Carboplatin ± MK1775	2	completed
NCT02196168	**Cisplatin With or Without WEE1 Inhibitor MK-1775 in Treating Patients With Recurrent or Metastatic Head and Neck Cancer**	Hypopharyngeal, Laryngeal, Lip and Oral Cavity, Nasal Cavity and Paranasal Sinus, Oropharyngeal, or Tongue Carcinoma	Cisplatin ± MK1775	2	terminated
NCT02101775	**Gemcitabine Hydrochloride With or Without WEE1 Inhibitor MK-1775 in Treating Patients With Recurrent Ovarian, Primary Peritoneal, or Fallopian Tube Cancer**	Ovarian Carcinoma;<br>Primary Peritoneal Carcinoma;<br>Fallopian Tube Cancer	Gemcitabine ± MK1775	2	recruiting
NCT01958658	**AZD1775, Cisplatin, and Radiation Therapy in Treating Patients With Cervical Cancer**	Cervical Cancer (Stage I B - III B)	MK1775 ± Cisplatin/Radiation	1	suspended
NCT02513563	**AZD1775 Plus Carboplatin-Paclitaxel in Squamous Cell Lung Cancer**	Squamous Cell Lung Cancer	MK1775 + Carboplatin/Paclitaxel	2	recruiting

NCT02037230	**Dose Escalation Trial of AZD1775 and Gemcitabine (+Radiation) for Unresectable Adenocarcinoma of the Pancreas**	Pancreatic Cancer	MK1775 + Gemcitabine/Radiation	1	recruiting
NCT02508246	**WEE1 Inhibitor MK-1775, Docetaxel, and Cisplatin Before Surgery in Treating Patients With Borderline Resectable Stage III-IVB Squamous Cell Carcinoma of the Head and Neck**	Head and Neck Squamous Cell Carcinoma	MK1775 + Cisplatin/Docetaxel	1	recruiting
NCT02194829	**Paclitaxel Albumin-Stabilized Nanoparticle Formulation and Gemcitabine Hydrochloride With or Without WEE1 Inhibitor MK-1775 in Treating Patients With Previously Untreated Pancreatic Cancer That Is Metastatic or Cannot Be Removed by Surgery**	Metastatic Pancreatic Adenocarcinoma; Pancreatic Cancer (Stage III-IV)	Pacitaxel/Gemcitabine ± MK1775	1 / 2	temporarily stopped
NCT01922076	**WEE1 Inhibitor AZD1775 and Local Radiation Therapy in Treating Younger Patients With Newly Diagnosed Diffuse Intrinsic Pontine Gliomas**	Diffuse Intrinsic Pontine Glioma; Untreated Childhood Anaplastic Astrocytoma, Anaplastic Oligoastrocytoma, Glioblastoma, or Gliosarcoma	Radiation + MK1775	1	recruiting

NCT01849146	**WEE1 Inhibitor AZD1775, Radiation Therapy, and Temozolomide in Treating Patients With Newly Diagnosed or Recurrent Glioblastoma**	Adult Glioblastoma;<br>Recurrent Glioblastoma	Radiation/Temozolomide + MK1775	1	recruiting
NCT01748825	**AZD1775 for Advanced Solid Tumors**	Neoplasms;<br>Lymphoma	MK1775	1	recruiting
NCT02341456	**Phase Ib Study AZD1775 in Combination With Carboplatin and Paclitaxel in Adult Asian Patients With Solid Tumours**	Advanced Solid Tumours	MK1775 vs.<br>MK1775 + Carboplatin vs.<br>MK1775 + Carboplatin/Paclitaxel	1	ongoing
NCT02087241	**Ph II Trial of Carboplatin and Pemetrexed With or Without AZD1775 for Untreated Lung Cancer**	Non-Squamous Non Small Cell Lung Cancer (Stage IV)	Carboplatin/Permetrexed ± MK1775	2	terminated
NCT02272790	**AZD1775 Plus Chemotherapy in Patients With Platinum-Resistant Ovarian, Fallopian Tube, or Primary Peritoneal Cancer**	Platinum-Resistant Epithelial Ovarian, Fallopian Tube, or Primary Peritoneal Cancer	MK1775 + Carboplatin vs.<br>MK1775 + Paclitaxel	2	recruiting
NCT01827384	**Molecular Profiling-Based Targeted Therapy in Treating Patients With Advanced Solid Tumors**	Advanced Malignant Solid Neoplasm;<br>Recurrent Malignant Solid Neoplasm	Veliparib + Temozolomide vs.<br>MK-1775 + Carboplatin vs.<br>Everolimus vs.<br>Trametinib	2	recruiting

NCT02666950	**WEE1 Inhibitor AZD1775 With or Without Cytarabine in Treating Patients With Advanced Acute Myeloid Leukemia or Myelodysplastic Syndrome**	Myelodysplastic/Myeloproliferative Neoplasm;<br>Adult Acute Myeloid Leukemia	Cytarabine ± MK1775	2	not yet recruiting
NCT02087176	**A Placebo Controlled Study Comparing AZD1775+ Docetaxel Versus Placebo+Docetaxel to Treat Lung Cancer**	Non Small Cell Lung Cancer	MK1775 + Docetaxel vs. Placebo + Docetaxel	2	terminated
NCT02688907	**Phase II, Single-arm Study of AZD1775 Monotherapy in Relapsed Small Cell Lung Cancer Patients With MYC Family Amplification or CDKN2A Mutation Combined With TP53 Mutation**	Small Cell Lung Cancer	MK1775	2	not yet recruiting
NCT02593019	**Phase II, Single-arm Study of AZD1775 Monotherapy in Relapsed Small Cell Lung Cancer Patients**	Small Cell Lung Cancer	MK1775	2	recruiting
NCT02381548	**Phase I Trial of AZD1775 and Belinostat in Treating Patients With Relapsed or Refractory Myeloid Malignancies or Untreated Acute Myeloid Leukemia**	Acute Myeloid Leukemia;<br>Chronic Myelogenous Leukemia (bcr-Abl1+)	MK1775 + Belinostat	1	recruiting

NCT02207010	**A Phase 0 Study of AZD1775 in Recurrent GBM Patients**	(Multiforme) Glioblastoma	MK1775	1	ongoing
NCT02585973	**Dose-escalating AZD1775 + Concurrent Radiation + Cisplatin for Intermediate/High Risk HNSCC**	Squamous Cell Carcinoma of Head and Neck	MK1775 + Cisplatin/Radiation	1	recruiting
NCT02791919	**Wee1 Kinase Inhibitor AZD1775 and Combination Chemotherapy in Treating Children, Adolescents and Young Adults With Relapsed or Refractory Acute Myeloid Leukemia**	Adult Acute Myeloid Leukemia;<br>Childhood Acute Myeloid Leukemia	MK1775 + Fludrabine/Cytarabine	1	Withdrawn
NCT02465060	**NCI-MATCH: Targeted Therapy Directed by Genetic Testing in Treating Patients With Advanced Refractory Solid Tumors, Lymphomas, or Multiple Myeloma**	Plasma Cell Myeloma;<br>Lymphoma;<br>Malignant Neoplasm;<br>Malignant Solid Neoplasm	MK1775*	2	recruiting

Table S1: Overview about current and already completed clinical trials with WEE1 inhibitor MK1775 (AZD1775) (last update 06/22/2017)

* only for patients with mutation in BRCA1 or BRCA2 gene
